# Deep learning analysis of particle content in extracted slow-release morphine: longer boiling reduces large fragments while retaining morphine extraction

**DOI:** 10.1038/s41598-026-35870-2

**Published:** 2026-01-19

**Authors:** Henrik Sahlin Pettersen, Per Ole M. Gundersen, Trond Oskar Aamo, Katrine Melby

**Affiliations:** 1 Department of Clinical and Molecular Medicine, The Norwegian University of Science and Technology, Trondheim, NO-7491 Norway; 2 Department of Pharmacology, St. Olavs Hospital, Trondheim University Hospital, Trondheim, NO-7030 Norway; 3 Department of Pathology, St. Olavs Hospital, Trondheim University Hospital, Trondheim, NO-7030 Norway; 4 Clinic of Substance Use and Addiction, St. Olavs Hospital, Trondheim University Hospital, Trondheim, NO-7030 Norway

**Keywords:** Morphine extraction, Slow-release tablets, Particle, Contamination, Harm reduction, Injecting drug use, Chemistry, Drug discovery, Health care, Medical research

## Abstract

**Introduction:**

Injecting drug users often extract morphine from slow-release oral tablets, potentially leading to harmful particle contamination upon injection. This study assesses the efficiency of morphine extraction and particle content of filtrates produced by various methods employed by drug users in Trondheim, Norway. The findings provide important insights that can inform harm-reduction services and healthcare providers in efforts to reduce injection-related morbidity among people who already inject drugs.

**Methods:**

Four extraction methods were evaluated using 60 mg Dolcontin tablets: Method A (no coating removal, 3-minute boiling), Method B (coating removal, crushing, 3-minute boiling), Method C (coating removal, 3-minute boiling), and Method D (coating removal, 10-minute boiling). Resulting solutions were filtered using cotton balls, and morphine content was quantified using LC-MS/MS. Particle content of filtrates was analyzed using slide scanning, deep learning-based particle segmentation, and QuPath image analysis software.

**Results:**

Morphine recovery ranged from 81.2% (Method D) to 91.3% (Method B). Method A yielded a significant presence of small insoluble particles (<100 μm), while Method B yielded the highest density of the largest particles (>500 μm). Method C exhibited the highest density of medium-sized particles (100-500 μm). Method D generated the fewest particles across all size categories.

**Conclusion:**

The extraction methods used by injecting drug users result in significant variability in morphine recovery and particle content of filtrates. Method D (10-minute vs. 3-minute boiling) demonstrated the highest efficiency in particle reduction, with only 10% less morphine recovery. Lack of coat removal significantly increases the number of primarily small (<100 μm) fragments. These findings highlight the importance of evidence-based harm-reduction measures to mitigate risks associated with injecting tablet-derived solutions. The results may support harm-reduction counselling and service design aimed at reducing particulate exposure and related complications, without endorsing or facilitating drug use.

## Introduction

Globally, an estimated 13.2 million people inject drugs as of 2021^1^. A systematic review and meta-analysis found that people who inject drugs have mortality rates nearly 15 times higher than their non-drug using peers, with an all-cause mortality rate of 2.9 per 100 person-years^2^. In Norway, injecting drug use accounts for approximately 80% of drug-related fatalities^3^. From 2002 to 2022, Norway experienced roughly 280 annual deaths related to use disorders, ranking it among the European nations with the highest per capita mortality rates from drug usage^4^. Health and medical interventions are needed beyond addressing overdoses and blood-borne infections like HIV and hepatitis C, particularly as globally about 600,000 deaths were attributable to drug use in 2019, with approximately 80% related to opioids^5^. Intravenous injection can cause blood vessel and vascular damage, potentially leading to conditions such as phlebitis, folliculitis, cellulitis, abscesses, and septicemia^6^.

While possession and use of drugs remains illegal in Norway, supervised injection facilities have been permitted since the 2004 Drug Injection Rooms Act. Recent policy discussions have focused on shifting from a crime-centered to a health-centered approach to drug use^7^. This research aligns with Norway’s harm reduction framework by studying ways to reduce injection-related health risks.

Understanding and studying drug preparation methods is fundamental to evidence-based harm reduction approaches. Recent research shows that injection-associated infections affect a majority of people who inject drugs, highlighting the urgent need to understand and address preparation practices^8^. This research follows established ethical frameworks centered on improving public health outcomes while acknowledging the complex realities faced by people who inject drugs^9^.

Bacterial infections often result from poor hand and skin hygiene, use of non-sterile or shared injection equipment, and contamination and toxicity of injected substances^10^. Despite the risks, injecting remains the preferred drug administration method for many opioid and stimulant users worldwide^11^.

To reduce particle contamination, drug users might use cigarette filters, cotton ball filters, or syringe filters before injection. However, concerns among users persist about drug loss during filtration (5, 6). Mclean et al.^12^, found that an unfiltered mixture from a slow-release morphine tablet contained millions of particles, some measuring ≥ 400 µm. Injecting such particles could cause significant harm (6). Cigarette filters substantially reduced the number of large particles, with cotton ball filters showing similar results but slightly lower morphine recovery^12^.

Effective harm reduction measures are urgently needed for this population. Opioid substitution therapy and syringe service programs are cost-effective strategies proven to lower the risk of HIV, hepatitis C, and other infections associated with opioid use. Further research on preventing opioid use-related infections, especially safer injection practices, is crucial^13–16^. The systematic study of drug preparation and injection practices serves a vital public health function. Research has shown that injection-related infections are widespread, affecting over 60% of people who inject drugs, demonstrating the critical importance of understanding and improving preparation practices^8^. By examining current practices in controlled settings, we can develop evidence-based interventions that minimize risks.

Machine learning and deep learning techniques have significantly enhanced pharmaceutical analysis through systematic processing of complex data^17^. Deep learning algorithms have proven valuable for pharmaceutical research^18^. Ensemble deep learning methods, which integrate multiple neural networks, have been effectively applied in bioinformatics to improve predictive performance and robustness^19^. Our study builds upon these advances by applying ensemble deep learning segmentation methods to quantify particle content in morphine extracts, integrating computational tools for evidence-based harm reduction research.

### Aim

This study aims to replicate and investigate various preparation techniques employed by substance users in Trondheim, Norway, building upon the work initiated by Mclean et al^12^. The primary focus is on the processing of slow-release oral morphine tablets, assessing the impact of each method on the residual morphine concentration in the filtered solution and the effectiveness in minimizing particle presence.

## Methods

### Morphine tablet composition

The morphine tablet evaluated in this study is Dolcontin (Mundipharma®, Mundipharma Corporation, Ireland), a 60 mg formulation containing 45 mg of morphine. The tablet’ s composition, as detailed in the product description, includes several additives with limited or no solubility in water: cetostearyl alcohol, magnesium stearate, talc, hydroxyethylcellulose, and macrogol 400. The presence of additional ingredients varies by tablet strength: anhydrous lactose is found in the 5 mg, 10 mg, 30 mg, and 60 mg versions, while hypromellose is present in the 10 mg, 30 mg, 60 mg, 100 mg, and 200 mg versions. Coloring agents also differ across strengths, with iron oxide (E 172) used in the 5 mg, 30 mg, 60 mg, and 100 mg tablets, and titanium dioxide (E 171) present in all strengths.

### Extraction methods evaluated

Using practices reported by injecting drug users in Trondheim, Norway, this study incorporated four prevalent methods for extracting morphine:

Method A: The tablet was brought to a boil with 6 ml of water for 3 minutes in a stainless steel cup, during which any floating coating material was removed as the tablet dissolved. Small volumes of water were added during boiling to achieve a final volume of approximately 5 ml.

Method B: The coating material was first removed using a wet cotton swab. The tablet was then crushed in a stainless steel cup using a pestle before boiling in 6 ml of water for 3 minutes. Water was added during boiling to obtain a final volume of approximately 5 ml.

**Figure 1:**
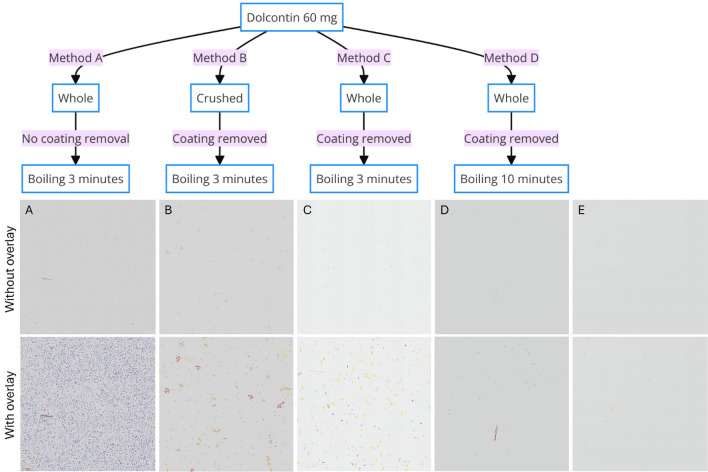
Flow chart (upper half) describing the different extraction Methods and quantitative image analysis (lower half). The upper scanned images A-E displays are without overlay, while the lower row of images shows the same slides segmentation overlay indicating segmentation by deep segmentation CNNs and measured particles and size groups: Less than 100 μm (blue), 100-250 μm (yellow), 250-500 μm (orange), and greater than 500 μm per mm² (red). Each column represents a different treatment condition as follows: (A) Dolcontin 60 mg whole tablets, no coating removal, boiling for 3 minutes (Method A). (B) Dolcontin 60 mg crushed tablets, coating removed, boiling for 3 minutes (Method B). (C) Dolcontin 60 mg whole tablets, coating removed, boiling for 3 minutes (Method C). (D) Dolcontin 60 mg whole tablets, coating removed, boiling for 10 minutes (Method D). (E) Control, no tablet, boiling for 3 minutes. All samples were subjected to cotton filtration. Particle segmentations resulted from an active learning deep learning-based segmentation using a dual CNN approach (DeepLabV3 with Resnet18 and Xception backbones).

Method C replicated the process of Method A, but the coating material was removed before boiling using a wet cotton swab. In Method D, the coating material was first removed using a wet cotton swab, and the tablet was then brought to a boil with 5 ml of water in a stainless-steel cup. The tablet was boiled for 10 minutes, and more water was added throughout to end up with a final volume close to 5 ml. The analysis included six tablets for Methods A and C, and three tablets each for Methods B and D (Fig1).

### Cotton ball filtration method

A cotton ball with a diameter of approximately 25 mm was divided into four equal parts and reformed into smaller balls. After cooling the boiled product, one ball was placed in the stainless-steel cup, swirled to absorb the liquid, and the product was drawn into a 5 ml syringe through the cotton. The filtrate was transferred to a 50 ml volumetric flask, with 100 µl kept for particle scanning. To extract morphine residues, 6 ml of water was added to the residue-containing cup, and the cotton ball was boiled for 5 minutes. This solution was drawn up via the cotton ball into a new 5 ml syringe and transferred to a 10 ml volumetric flask. Blank samples underwent the same procedure to identify any particles introduced during filtration.

### Morphine content analysis

For morphine content analysis, the contents of both the 50 ml and 10 ml volumetric flasks were diluted with 0.5% acetic acid. From each flask, a 1 ml aliquot was filtered through a 0.45 µm membrane into individual vials. This membrane filtration step was performed only for LC-MS/MS sample preparation and was not intended to model syringe-filter use in real-world injection settings; particle scanning was performed on aliquots of the cotton-filtered extract. Subsequently, 25 µl of this filtrate was diluted with 25 ml of MeOH, and 25 µl internal standard (20 µg/ml morphine-d3) was added. To comply with an LC-MS/MS Method developed for morphine quantification in urine by Hegstad et al., a 1000-fold final dilution was required^20^. A 0.2 µl aliquot was injected on an Acquity UPLC I-class coupled to a Waters Xevo TQ-S tandem-quadrupole mass spectrometer (Waters Corp., Milford, MA, USA). Chromatographic separation was achieved on an Acquity UPLC HSS T3 column (2.1 × 100 mm, 1.8 μm) with an Acquity UPLC HSS T3 VanGuard pre-column (2.1 × 5 mm, 1.8 μm), using gradient elution with a mobile phase consisting of methanol and 0.1% formic acid in water. The Xevo TQ-S was operated in positive electrospray ionization (ESI) mode with multiple reaction monitoring, employing mass transitions m/z 286.1 > 201.0 and m/z 286.1 > 165.0 for morphine quantification and qualification. The mass transition m/z 289.0 > 201.0 was used for detecting morphine-d3. Morphine had a retention time of 0.91 minutes. Morphine HCl standards (Lipomed, Arlesheim, Switzerland) were prepared in methanol at concentrations of 0.01 µg/ml, 0.05 µg/ml, 0.10 µg/ml, 0.50 µg/ml, and 2.00 µg/ml. Limit of Detection (LoD) and Limit of Quantification (LoQ) were 6 µg and 20 µg morphine per tablet, respectively. Pairwise comparisons between methods (A, B, C, and D) for extracted morphine, remaining morphine, and recovery efficiency were performed using a two-tailed, independent (unpaired) Student’s t-test, assuming unequal variances (Welch’ s t-test). The statistical analysis was conducted in Python using the scipy.stats.ttest_ind function.

### Particle quantification using deep semantic segmentation

Particle quantification was performed on scanned brightfield microscopy slides using QuPath software version 0.3.2. The analysis employed a multi-step digital image analysis pipeline. First, artifact detection was performed using pixel intensity-based thresholding to identify air bubbles and edge disturbances. Subsequently, QuPath’ s built-in ’ Train object classifier’ tool was used with a two-class random trees pixel classifier to differentiate between valid fluid areas and artifacts. This random forest classifier was trained on manually annotated regions of interest (ROIs) and used intensity, texture, and morphological features to segment the image into analyzable regions and artifacts. The classifier was iteratively trained on at least 20% of slides, particularly from challenging areas, and then applied to the remaining unannotated slides.

Manual annotation of particles was initially performed on approximately 10% of the slides using QuPath’ s thresholding functionality. Patches from annotated and unannotated slides were exported for deep learning-based particle segmentation using the active learning pipeline described in NoCodeSeg. Two DeepLabV3 networks with Resnet18 and Xception backbones were trained on approximately 800 patches of size 2048x2048 with a downsampling factor of four. Unannotated patches were iteratively annotated using DeepMIB^21,22^, prioritizing patches with high Intersection over Union (IoU) agreement between the two networks. The scripts used are available in the NoCodeSeg repository^23^.

The fully annotated patches with particle annotations were imported back into QuPath. Annotations were converted to detections for size assessment and classification of particles into four size categories: under 100 μm, 100-250 μm, 250-500 μm, and over 500 μm. Detected sizes were hierarchically incorporated as detections within valid liquid areas, excluding air bubbles and glass artifacts for accurate particle concentration calculations. The generated CSV files were processed in Matplotlib, and statistically significant differences between boiling Methods within each particle size category were assessed using an ANOVA test for P-value calculation.

## Results

Morphine recovery levels varied notably across different methodologies, with mean values ranging from 31.3 to 32.5 mg and standard deviations between 2.3 and 12.9. A comprehensive analysis, considering the morphine quantities in both the filtrate and cotton balls, revealed a recovery efficiency of 81.2% to 91.3% (Table 1).

**Table 1:** Results from the analysis of morphine content using the different extraction methods (A, B, C and D) (n = number of parallels)

Method	n	Extracted morphine from cotton ball (mg)	Standard deviation (mg)	Extracted remainings from cotton ball (mg)	Standard deviation (mg)	SUM (mg)	Morphine recovery efficiency (%)
A	6	32.5	13.2	7.7	1.8	40.2	89.3
B	3	31.8	2.4	9.2	1.8	41.1	91.3
C	6	32.4	12.9	7.6	1.1	40.0	88.8
D	3	31.3*	2.3	5.2**	0.8	36.5	81.2***

The table shows the average extracted morphine from the cotton ball, the standard deviation of the extracted amount, the average morphine remainings left in the cotton ball after extraction, the standard deviation of the remainings, the total morphine recovered (sum of extracted morphine and remainings), and the overall morphine recovery efficiency percentage for each method. Statistical differences between methods were evaluated using Student’ s t-test for each column, where * indicates p<0.05, ** indicates p<0.01, and *** indicates p<0.001.

Statistical analysis revealed significant differences between extraction methods. Method D showed significantly lower morphine recovery (81.2%) compared to Methods A (89.3%, p<0.05), B (91.3%, p<0.01), and C (88.8%, p<0.05). However, the absolute difference in extracted morphine between Method D and other methods was relatively small (1.1-1.2 mg), suggesting that the longer boiling time maintains clinically relevant extraction efficiency while reducing particle contamination. Method B showed the highest total morphine recovery (41.1 mg ± 2.4), significantly higher than Method D (36.5 mg ± 2.3, p<0.001), likely due to the mechanical disruption of the tablet structure during crushing.

Sample-to-sample variability was quantified using standard deviation (SD). Methods A and C showed higher variability in morphine extraction (SD = 13.2 mg and 12.9 mg, respectively) compared to Methods B and D (SD = 2.4 mg and 2.3 mg, respectively). This increased variability in Methods A and C may be attributed to inconsistencies in the natural dissolution process without mechanical disruption.

The particle content analysis workflow is outlined in Figure 1, showing both the extraction methods and the deep learning-based segmentation approach used for quantification. Method A yielded an average morphine recovery of 32.5 mg, increasing to 40.2 mg when including the residues from the cotton balls. The filtrate contained numerous black dots, likely insoluble coating material from the tablets, with minimal crystal presence and mostly small fragments across all Methods. Method B, involving tablet coating removal and crushing, resulted in a morphine recovery of 31.2 mg, increasing to 41.1 mg when including the cotton ball content.

**Figure 2:**
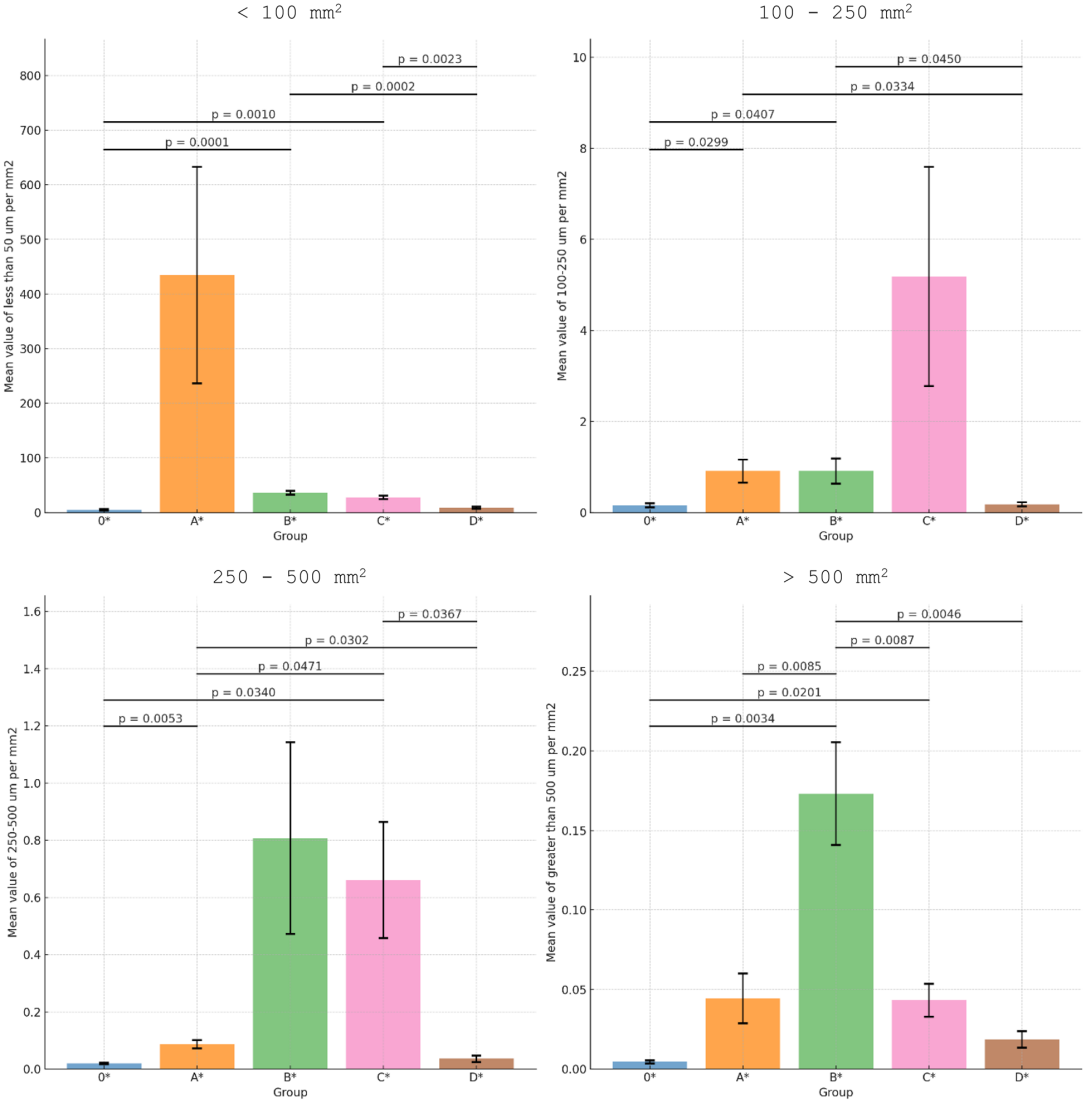
Comparison of group differences in mean vessel densities across different particle size categories. Four subfigures represent different vessel diameter ranges: less than 100 μm (top left), 100-250 μm (top right), 250-500 μm (bottom left), and greater than 500 μm per mm² (bottom right). Each subfigure displays mean values with standard error bars for five different groups (pretreatments) Method O* (control); Method A*: No coating removal and 3-minute boiling; Method B*: Removing the coating, crushing, and boiling for 3 minutes; Method C*: Removing the coating and boiling for 3 minutes; Method D*: Removing the coating and boiling the tablet for 10 minutes. Statistically significant differences between the different pretreatment groups are denoted by lines and respective p-values.

**Figure 3:**
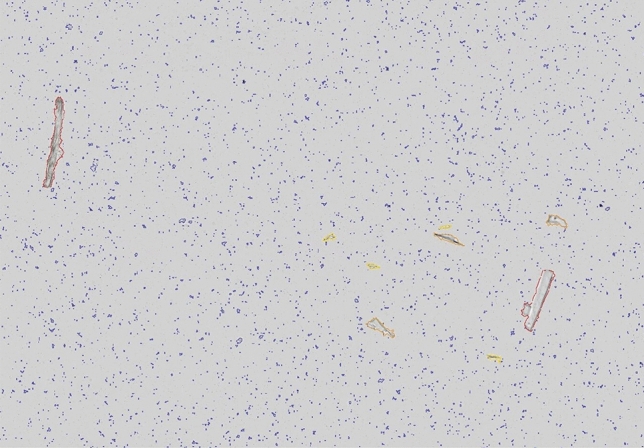
Example image of final particle segmentations resulting from DeepMIB active learning processing with DeepLabV3 networks with Resnet18 and Xception. The image shows the result after fully annotated patches with particle annotations were imported into QuPath and annotations were then converted to detections for classification of particles into four distinct groups. Less than 100 micrometers (blue), 100 to 250 micrometers (yellow), 250 to 500 micrometers (orange), and greater than 500 micrometers (red).

Particle analysis showed that Method A had the highest mean density of the smallest particles (<100 μm), while Method B had the greatest density of the largest particles (>500 μm). Method C, which involves the removal of the coating and boiling of the tablet, exhibited the highest density of medium-sized particles (100-500 μm). In contrast, Method D showed the fewest particles across all size categories (Figure 2). These size distributions were quantified using our deep learning segmentation approach, which classified particles into four distinct size groups (Figure 3).

Comparative analysis with previous findings^12^ revealed some important differences in particle size distributions. McLean et al. reported particles ranging from <5 μm to >400 μm in unfiltered preparations, with cigarette filters effectively removing most particles >50 μm. Our study found particles extending past this range (>500 μm), particularly in Method B (crushing). The enhanced detection of larger particles in our study may be attributed to our use of deep learning-based segmentation which enabled more systematic detection across the full-size spectrum. The proportion of particles <100 μm remained substantial in all our preparations, with Method A showing particularly high counts (mean density: 245 ± 34 particles/mm²), emphasizing the importance of additional filtration beyond cigarette filters.

Microscopic examination of the particle content revealed partly small (< 100 μm) uncharacteristic black circular fragments, or medium to large sized (100-500 μm) either crystal-like elongated fragments or more ”cotton like” circular fragments, as demonstrated in the segmentation results (Figure 3).

## Discussion

All preparation methods yielded a morphine extraction above 80%. Considering both the extraction of morphine and the particle size distribution, Method D, followed by Method A, proved most effective in our study. Significant variability was noticed in morphine extraction rates and particle sizes within iterations of the same method. Compared to a blank sample, all methods showed a marked difference in particle content.

Mclean et al.^12^, reported morphine sulfate recovery exceeding 90%, accommodating a 5% variability in content between tablets (57-63 mg). In their research, morphine sulfate was retained by all filters but could be recovered through subsequent washes. Our analysis assessed the morphine content from a total of 45 mg in a 60 mg tablet, yielding an extraction efficiency of approximately 80-90%, when including the remaining material of the cotton ball.

Mclean et al.^12^ demonstrated that unfiltered extracts from tablets contained particle sizes ranging from <5 μm to >400 μm, with cigarette filters removing particles >100 μm. Our study found particle sizes from <100 μm to >500 μm, systematically grouped into four categories (figure 1 and 2). The tablet compression using a pestle and mortar could yield denser and consequently larger particles, as observed in Method B, unlike the dissolution of tablets through boiling as performed in Methods A, C, and D. Prolonged boiling in Method D might account for the smaller particle sizes observed. Method D also showed modestly lower total morphine recovery. One possible contributor is chemical degradation during heating; morphine degradation in aqueous solutions has been described, with the extent influenced by oxygen exposure and pH^24^. Because we did not quantify known degradation products (e.g., pseudomorphine, morphine-N-oxide), we cannot exclude degradation as a contributor. However, the protocol involved repeated water addition to maintain a final volume close to 5 ml, and the reduced recovery may also reflect non-degradation mechanisms such as adsorption/retention on vessel surfaces, altered dissolution/filtration dynamics, or losses during transfers. Future time-course experiments quantifying both morphine and degradation products would help distinguish these mechanisms. The scanned regions revealed crystals, black dots, and other particle types.

Mclean et al.^12^, noted that hot extraction techniques for morphine tablets risk wax passing through filters or cotton balls, which can solidify upon cooling, forming particulate matter and potentially resulting in higher filtrate contamination compared to cold extraction methods (not evaluated in our study).The particle size distributions observed in this study have critical clinical implications. Particles greater than 500 μm, predominantly found in Method B, pose a heightened risk of vascular occlusion and localized inflammation upon injection. Research indicates that intravenous administration of particulate matter can lead to mechanical blockage of blood vessels and activate clotting mechanisms, resulting in vascular occlusion and inflammation^25^. In contrast, the substantial reduction in particle counts and sizes observed with Method D in our experimental setup suggests that longer heating duration may reduce particulate burden in tablet-derived extracts. These findings may support evidence-based harm-reduction counselling and service design delivered by healthcare and harm-reduction services, without endorsing or facilitating drug use. This could potentially mitigate risks of septicemia and endocarditis, as people who inject drugs are at a significantly higher risk of developing these conditions compared to the general population^26^.

The removal of an iron oxide coating, primarily in the initial preparation phase of Methods B, C, and D, and post-boiling in Method A, likely caused the significant presence of insoluble particles (black dots) identified in Method A. The detected crystals are hypothesized to be morphine, while particles >500 μm could be cotton fragments (figure 1 and 2).

We selected scan-based image analysis because it provides particle morphology and supports systematic detection of infrequent large fragments in addition to size binning and particle density estimates. Conventional particle sizing methods such as laser diffraction are rapid for bulk suspensions but typically require assumptions about optical properties and provide limited information about particle identity; in our setting the filtrates contain heterogeneous material (tablet excipients/coating, crystals, and fibers) and low-volume extracts (approximately 5 ml), making direct morphology and particle-type differentiation particularly relevant. Future work could complement our approach with laser diffraction or dynamic light scattering to enable cross-method validation and reporting of distributional summary metrics (e.g., polydispersity).

Several limitations were identified in the current study, including the crystallization observed in samples after an extended period. To minimize the time between tablet preparation and scanning, the three samples from each preparation Method were scanned consecutively. However, some samples were left for a longer time due to the scanning duration, and Methods A and C were prepared and analyzed in separate batches. This procedure could contribute to variations in sedimentation and crystallization observed within the same method. Additionally, disparities in particle content between batches have been noted, consistent with findings from McLean et al^12^.

Repeated tests revealed that Method B, which involved crushing the tablets, led to more crystallization compared to boiling the tablet whole in Methods A, C, and D. The variability observed between the two batches may mirror real-world conditions where individuals lack access to sterile equipment and methods for extracting morphine. Drug users often resort to improvised techniques, such as crushing tablets between two spoons. However, this study implemented a reproducible method in a sterile setting, likely leading to enhanced morphine recovery and the production of fewer large particles than would occur outside a laboratory setting. In addition, we did not experimentally evaluate membrane syringe filters (e.g., 0.22 $\mu$m or 0.45 $\mu$m) for their effects on particle burden and morphine retention; McLean et al. demonstrated significant additional particle reduction using such syringe filters^12^, and we would expect a similar effect on our samples. Still, the availability and acceptability of such filters may be limiting for adoption. Finally, while we tested individual preparation variables (coating removal, crushing, boiling duration), not all combinations were systematically evaluated (e.g., coating removal combined with crushing and extended boiling); factorial experiments could assess such combinations to optimize the balance between morphine recovery and particulate reduction.

This study demonstrates that the common practices employed by injecting drug users for morphine extraction result in significant variability in both the amount of morphine extracted and the size of particles produced. In Norway, injecting the drug remains a prevalent administration route compared to other European countries. This can be due to the high cost and limited availability of the drug in Norway, as it is geographically located at the far end of drug transportation routes. To address these issues, several measures have been implemented, such as an opioid maintenance treatment program^3^. Other measures have been establishment of supervised injection sites or drug consumption rooms provides users with sterile injection equipment and a safe environment for consumption^13^, as well as providing drug checking services of drugs for people who use drugs in order to educate users on safe drug use^27^.

This research approach follows established ethical frameworks in harm reduction research^28^, where the goal is not to promote or enable drug use, but rather to reduce morbidity and mortality among individuals who already inject drugs. Understanding the technical aspects of preparation methods allows healthcare providers and harm reduction programs to give evidence-based advice that can prevent serious health complications while maintaining appropriate ethical boundaries.

Dissemination of these findings should occur primarily through established harm-reduction and clinical channels (e.g., supervised consumption services/drug consumption rooms, syringe service programs, and addiction treatment providers), ideally using co-developed educational materials that emphasize infection prevention, safer injection support, and access to treatment. This service-mediated approach helps translate evidence into reduced harm while avoiding user-facing procedural guidance.

Injecting drug use remains the preferred mode of administration among many opioid and stimulant users worldwide, despite the associated negative consequences, including increased risk of overdose, blood-borne infections, and social stigma^4^. Hanoa et al conducted a qualitative study that identified several factors contributing to the preference for injecting, such as cost-effectiveness, social interaction and peer learning, rapid and intense intoxication, and the ritual aspects of the injection process itself^11^. To address the urgent need for effective harm reduction measures in addition to available treatment, prevention strategies for related morbidity should include the provision of proper injection equipment, safe consumption environments, and education on safe drug use practices^11,16^.

## Conclusion

This study underscores the critical importance of evidence-based harm reduction strategies, particularly given that people who inject drugs face premature death and additional risks of serious complications from injecting tablet-derived solutions containing harmful particles. By systematically analyzing common preparation methods for morphine extraction, our findings demonstrate that longer boiling times significantly reduce particle contamination while maintaining reasonable morphine recovery, whereas lack of coating removal leads to increased small particle contamination. These findings have the potential to inform and reduce injection-related complications among people who inject drugs. To ensure these insights translate into practice without encouraging drug use, dissemination should occur primarily through established harm-reduction and clinical channels (e.g., supervised consumption services and addiction healthcare services), empowering staff to provide evidence-based counselling and to connect individuals to treatment and preventive care.

## Data Availability

The scanned slides, raw MS-data and segmentation masks for the particle content analyses are available from the corresponding author upon reasonable requests.
